# Metabolic plasticity supports a flexible nutritional symbiosis in *Cardiocondyla* ants

**DOI:** 10.1038/s41467-026-76801-z

**Published:** 2026-08-21

**Authors:** Phoebe Cunningham, José M. Martín-Durán, Jan Oettler, Eva Schultner, Rayko Halitschke, Tobias Engl, Martin Kaltenpoth, Lee M. Henry

**Affiliations:** 1 School of Biological and Behavioural Sciences, Queen Mary University London, London, United Kingdom; 2 Zoology and Evolutionary Biology, University Regensburg, Regensburg, Germany; 3 Mass Spectrometry and Metabolomics, Max Planck Institute for Chemical Ecology, Jena, Germany; 4 Department of Insect Symbiosis, Max Planck Institute for Chemical Ecology, Jena, Germany

**Keywords:** Coevolution, Microbial ecology, Transcriptomics, Molecular ecology

## Abstract

Nutritional symbioses have repeatedly evolved in insects, yet how hosts regulate these partnerships to balance benefits across development and environments remains unclear. Ants provide an exceptional system to address this question, as several lineages maintain ancient symbionts that can be naturally lost without harming the host. The invasive tramp ant *Cardiocondyla obscurior* provides a compelling example of this phenomenon, as the vertically transmitted symbiont *Candidatus Westeberhardia cardiocondylae* can be lost at the species, colony, and even individual level. By implementing a range of molecular techniques, including dual-RNA sequencing, fluorescent in-situ hybridisations of RNA and qPCR, in conjunction with experimental manipulations of diet, we reveal the regulatory dynamics and function of this labile association. Symbionts provide shikimate-derived nutrients that enhance colony resilience under protein limitation, while hosts appear to regulate symbiont abundance during protein scarcity and cuticle formation to optimise resource allocation. In the symbiont’s absence, ants compensate by upregulating genes enabling tyrosine acquisition from external sources. This metabolic flexibility allows colonies to exploit symbiont-derived and environmental nutrients, sustaining both growth and survival across nutritional contexts. Our findings reveal dynamic host regulation as a mechanism for sustaining the persistence and adaptability of ancient symbioses.

## Abstract (web summary)

Ancient symbioses may enable ants to thrive in diverse niches, but how these relationships are regulated remains unclear. This study reveals that some ants can dynamically regulate symbiont-derived benefits depending on the environmental context.

## Introduction

Acquiring sufficient nutrients is a central challenge for insects, particularly for social species where dietary limitations can impact both individual and colony-level fitness. Nutrient-provisioning symbioses have repeatedly evolved as a solution, enabling insects to exploit nutrient-imbalanced diets and colonise specialised niches such as obligate sap- or blood-feeding^1–5^. However, symbioses are not without cost. They are metabolically demanding, and hosts must balance the energetic investment of maintaining symbionts against their nutritional benefits^6^. Studies have shown that insects can regulate symbionts through various mechanisms, such as adjusting symbiont density, biasing them to specific developmental castes or sexes, or recycling them when their function is complete^7–11^. Yet, in most systems, how hosts regulate symbiont growth and coordinate metabolism to optimise benefits remains poorly understood.

Ants offer unique and underexplored models to address this question. At least four ant lineages have independently evolved intimate associations with *Sodalis*-allied bacteria—strictly vertically transmitted, intracellular symbionts housed in specialised host cells called bacteriocytes^12–14^. Despite their ancient origin and localisation within host-derived organs, we find these symbioses are remarkably labile: in some host lineages, symbionts are entirely absent from queens and even whole colonies in nature^12,13^. The evolutionary persistence of such flexible associations remains a puzzle. One hypothesis is that ants dynamically regulate their symbionts to meet nutritional demands, although the mechanisms by which this may occur remains unknown.

Genomic evidence suggests that *Sodalis*-allied symbionts in ants have all retained a conserved capacity to synthesise tyrosine via the shikimate pathway^13^. Tyrosine is essential for cuticle formation, melanisation, and immune function, but can only be obtained from protein (directly or via phenylalanine), making it limiting in protein poor niches such as arboreal habitats^15–17^. High carbohydrate-to-protein diets can also severely constrain ant colony growth^18^. We therefore hypothesise that in environments with inconsistent protein availability, tyrosine is a key limiting resource for ants, selecting for alternative supplies of this amino acid, including symbiont-derived sources.

Here, we use the metabolically streamlined endosymbiont *Westeberhardia* in *Cardiocondyla obscurior* to uncover how hosts regulate symbionts in response to nutritional demands, and to confirm that the functional role of the symbiont is to maintain colony productivity when protein is limited. Arboreal *C. obscurior* provides an ideal model, combining experimental tractability with natural variation in symbiont presence in queens and entire colonies^12,19,20^. Through dietary manipulation and multi-omics analysis of symbiotic and aposymbiotic colonies, we show that *Westeberhardia* enhances colony fitness under protein scarcity via tyrosine and folate provisioning, and that the host flexibly regulates its metabolism and appears to influence symbiont density in response to its diet. Our findings highlight the molecular basis of host–symbiont coordination and offer insights into how a nutritionally important, yet evolutionarily labile, symbiosis can persist over millions of years.

## Results

### Symbiont improves colony productivity under protein starvation

**Fig. 1:**
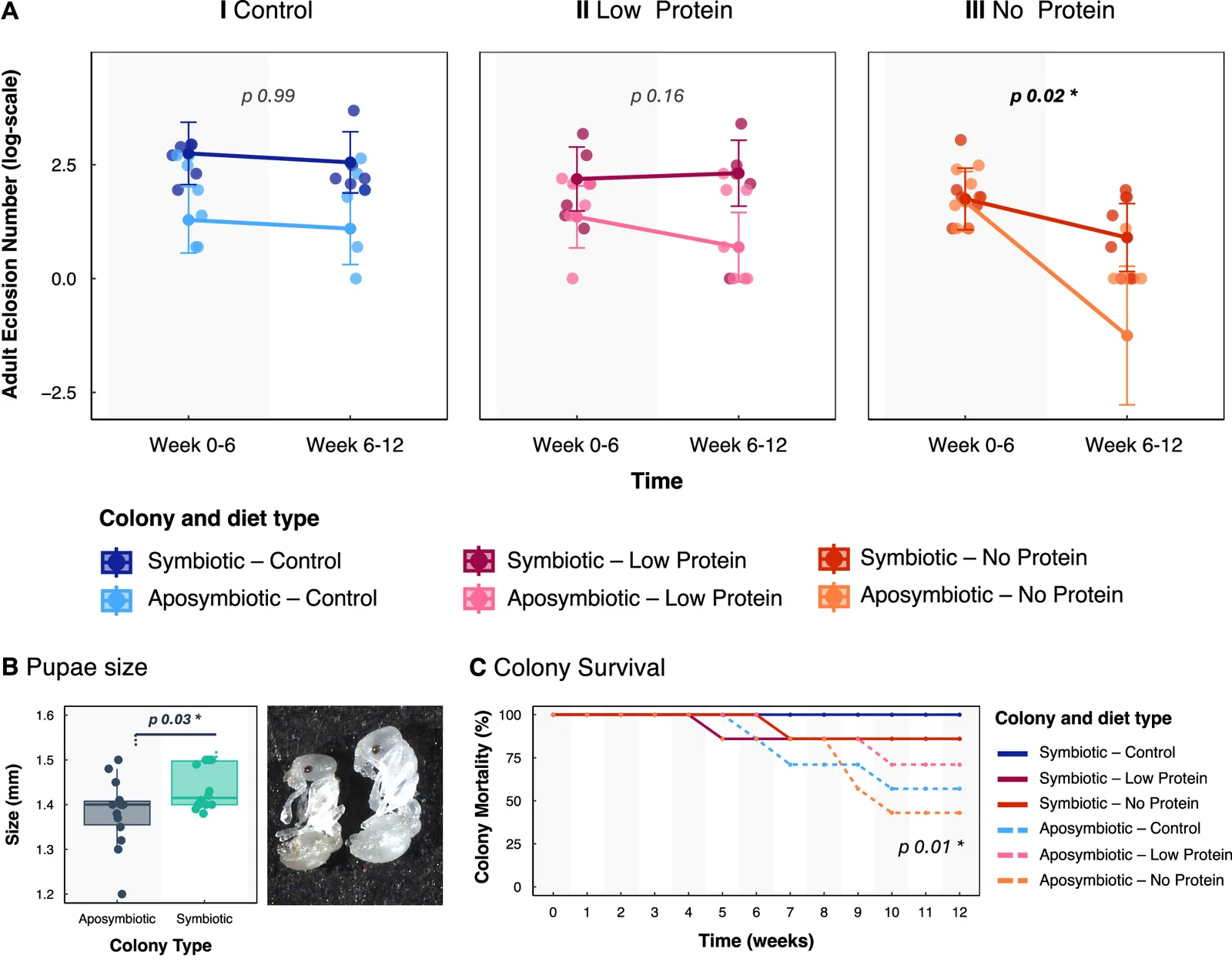
Effects of symbiont removal and protein starvation on colony and individual fitness. **A** Adult eclosion under control (dark/light blue), low-protein (dark/light pink), and no-protein (dark/light orange) diets in symbiotic vs. aposymbiotic colonies. Significance was assessed using generalised linear mixed-effects models fitted with a negative binomial distribution and colony identity as a random effect, followed by Tukey-adjusted two-sided Wald *z* tests for pairwise comparisons (*n* = 7 biological replicates per group, raw data points are presented along with mean values +/- SEM); (**B**) Pupae size in symbiotic vs aposymbiotic colonies on control diets tested statistically using a two-sided Welch’s *t* test. Aposymbiotic pupae size (*n* = 13 biological replicates, minima 1.30, maxima 1.48, median 1.40, Q1 1.36, Q3 1.41), symbiotic pupae size (*n* = 13 biological replicates, minima 1.38, maxima 1.50, median 1.42, Q1 1.50, Q3 1.50); (**C**) Colony survival across protein treatments, *p* value based on Cox proportional hazards model with a Tukey-adjusted two-sided Wald *z* test and represents significant difference between colony types (*n* = 7 starting biological replicates). All model outputs are in Supplementary Data 1. Asterisks denote significance on a scale: padj <0.05 *, padj <0.005**, padj <0.0005 ***.

To test the functional role of *Westeberhardia*, we simulated natural protein deprivation by feeding symbiotic and aposymbiotic *C. obscurior* colonies diets with reduced protein levels based on their standardised diet. Protein deprivation disproportionately affected later developmental stages in aposymbiotic colonies. Under complete protein starvation, pupal production in aposymbiotic colonies declined markedly, approaching significance (z = −1.79, *p* = 0.07; Fig. S1A). This effect intensified at eclosion, where adult emergence dropped by 94% in aposymbiotic colonies compared to only 57% in symbiotic colonies (z = −2.38, *p* = 0.02; Fig. 1A), underscoring the critical role of symbiont-derived resources in supporting development under protein scarcity. We also found that under optimal dietary conditions, symbiotic colonies were able to produce larger workers (*t*(22.1) = –2.3, *p* = 0.03; Fig. 1B) and exhibited a 75% lower risk of colony mortality, (*HR* = *0.25, p* = *0.01*, 95% CI: 0.081 – 0.79, Fig. 1C). Notably, worker cuticle thickness did not differ between groups, regardless of diet (Fig. S2). These results suggest *Westeberhardia* can aid in sustaining brood production during periods of protein deficiency, while maintaining colony health, and fortify worker size, under optimal conditions.

### Symbiont density is dynamically regulated across host development and diet

**Fig. 2:**
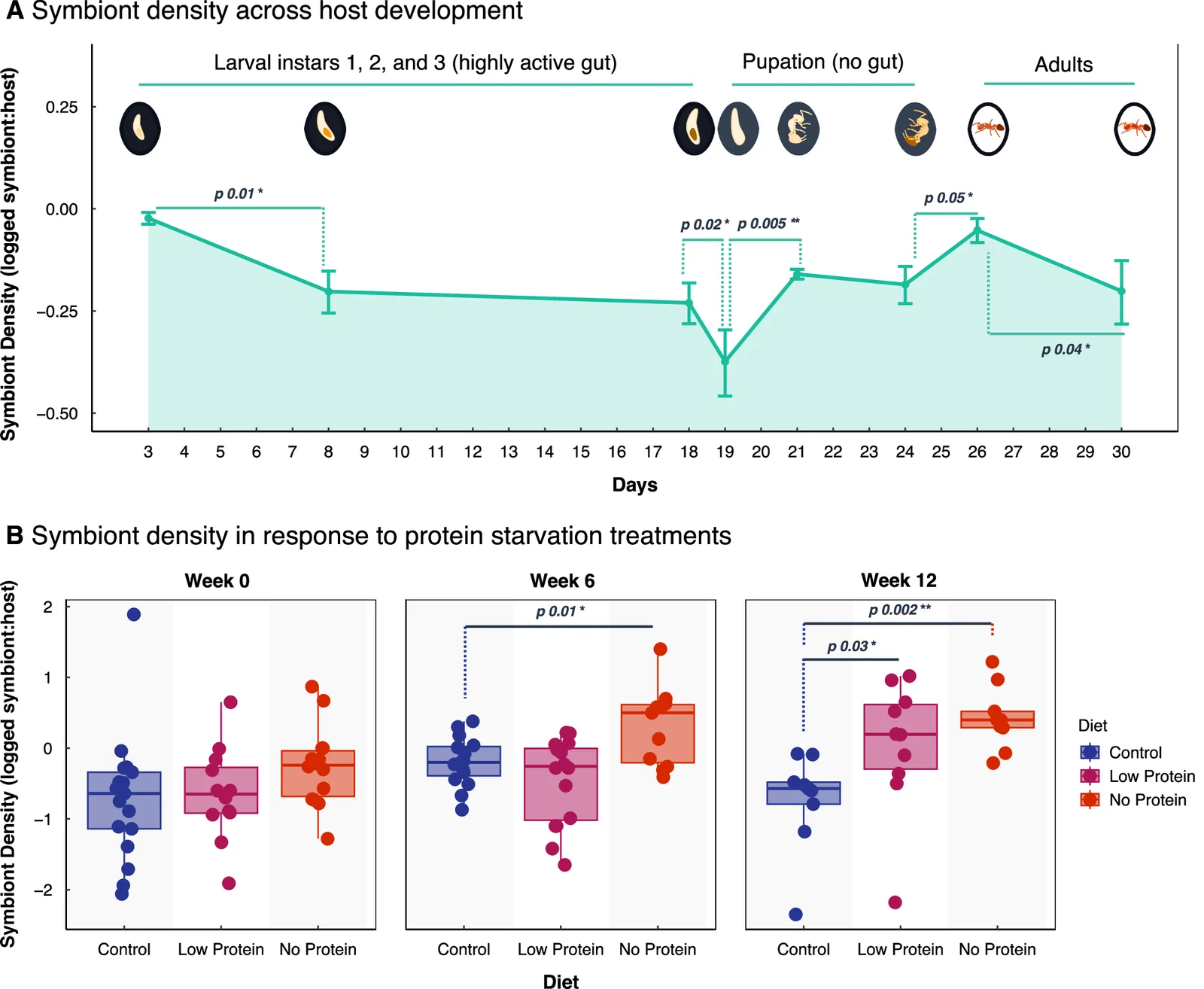
Symbiont density across host development and in response to protein starvation. Symbiont density, measured as the ratio of symbiont gene ribonucleoside-diphosphate reductase 1 subunit beta (nrdB) to host gene elongation factor-1 (EF-1) shown (**A**) across development stages (differences between life stages were tested using linear regression analysis followed by Tukey-adjusted two-sided Wald *z* tests for pairwise comparisons, *n* = 9 biological replicates per stage, data are presented as mean values +/- SEM); and (**B**) in response to no-protein (dark orange), low-protein (magenta), and control (blue) diets at weeks 0, 6 and 12. Differences were determined by linear regression analysis using two-sided *t* tests derived from the linear model coefficients. No multiple-comparison correction was applied. Control - week 0 (*n* = 17 biological replicates, minima −2.06, maxima 1.89, median −0.64, Q1 −1.14, Q3 −0.34), low-protein - week 0 (*n* = 12 biological replicates, minima −1.91, maxima 0.65, median −0.65, Q1 −0.92, Q3 −0.27), no-protein - week 0 (*n* = 14 biological replicates, minima −1.28, maxima 0.87, median −0.24, Q1 −0.68, Q3 −0.04), control - week 6 (*n* = 15 biological replicates, minima −0.87, maxima 0.38, median −0.20, Q1 −0.39, Q3 0.25), low-protein - week 6 (*n* = 16 biological replicates, minima −1.65, maxima 0.22, median −0.26, Q1 −1.02, Q3 0.00), no-protein - week 6 (*n* = 11 biological replicates, minima −0.41, maxima 1.4, median 0.5, Q1 −0.26, Q3 0.62), control - week 12 (*n* = 9 biological replicates, minima −2.35, maxima −0.08, median −0.57, Q1 −0.79, Q3 −0.48), low-protein - week 12 (*n* = 10 biological replicates, minima −2.18, maxima 1.02, media0.20, Q1 −0.30, Q3 0.62), no-protein - week 12 (*n* = 9 biological replicates, minima −0.21, maxima 1.22, median 0.4, Q1 0.29, Q3 0.52). Asterisks denote significance on a scale: padj <0.05 *, padj <0.005**, padj <0.0005 ***.

We found that symbiont density varied dramatically across development, dropping sharply at the pre-pupal stage (~day 18–19, t(15) = 2.48, *p* 0.02; ~day 19–21, t(15) = −3.26, *p* 0.005) after the ant has voided its meconium, which may disrupt gut-localised bacteriocytes (Fig. 2A). We then see a dramatic 1.6-fold recovery of symbiont density over 1-2 days when the pupa begins to form the morphology of an adult (Fig. 2A, days 19–28). This rapid rebound could indicate a host-assisted symbiont recovery during a key developmental transition when the adult cuticle is forming, a process the symbiont appears to be critical for.

We also found that symbiont density responded to dietary stress. Colonies deprived of protein showed significantly increased symbiont density by week 6 (*F*(39) = 7.06, no protein *p* = 0.02), and by week 12, colonies on both the low and no protein diets had significantly increased symbiont densities (*F*(25) = 5.99, low protein *p* = 0.03, no protein *p* = 0.002; Fig. 2B). These findings suggest an adaptive host response that may promote symbiont growth during key developmental stages and when exogenous protein is limited.

### Host transcription supports symbiont recovery at key developmental stages

**Fig. 3:**
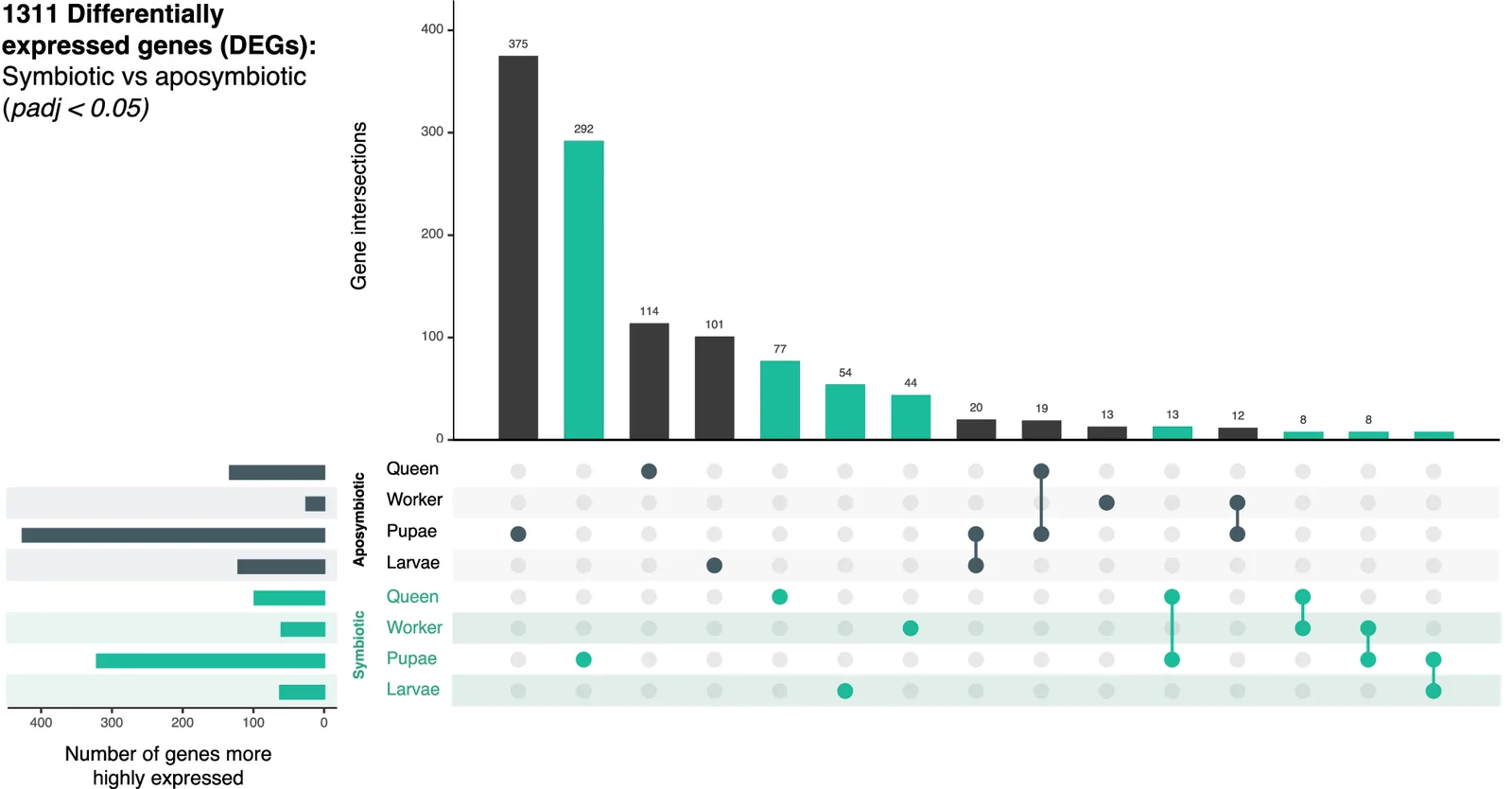
Differential gene expression between symbiotic and aposymbiotic colonies. Upset plot highlighting genes more highly expressed in one condition relative to the other, with genes more highly expressed in symbiotic colonies shown in turquoise and genes more highly expressed in aposymbiotic colonies shown in grey, across life stages or castes. Connected points indicate DEGs shared across multiple comparisons, representing a small subset of total DEGs (*n* = 3 biological replicates per life stage or caste). Asterisks denote significance on a scale: padj <0.05 *, padj <0.005**, padj <0.0005 ***.

Dual RNA-seq comparison of symbiotic versus aposymbiotic individuals (whole bodies) revealed over 650 differentially expressed genes (DEGs) at the pupal stage—far greater than at any other stage or caste (Fig. 3). By contrast, no DEGs were detected in *Westeberhardia* across host developmental stages (Fig. S3). These findings support our hypothesis that the symbiont plays a particularly important role during pupal development and that the principal method of symbiont regulation, e.g., population growth, is through host-derived mechanisms.

**Fig. 4:**
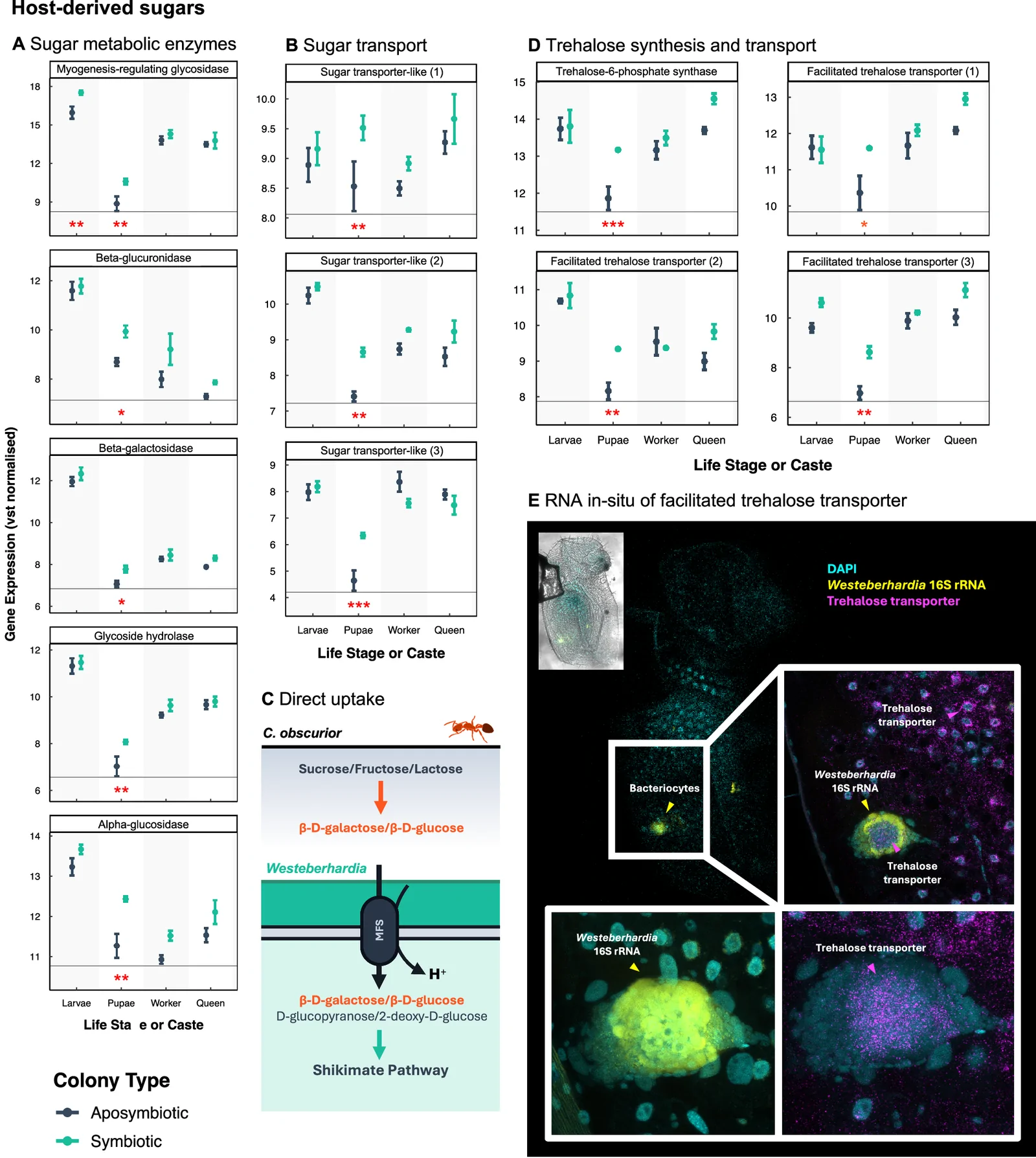
Expression patterns of putative host-symbiont regulatory genes. **A** Expression of host genes for enzymes to convert sucrose, fructose, or lactose into β-D-galactose/β-D-glucose; and (**B**) sugar transporters, are upregulated in symbiotic colonies; **C** Schematic showing *Westeberhardia* can uptake these sugars for use in the shikimate pathway; **D** Trehalose synthesis and transport genes show increased expression in symbiotic colonies (*n* = 3 per life stage or caste in all gene expression analyses, data are present as mean values +/- SEM); **E** Trehalose transporter shows concentrated expression in symbiotic larval bacteriocytes. For panels (**A**), (**B**), and (**D**), differential gene expression analysis was performed using DESeq2 in R with negative binomial generalised linear models. Significance was assessed using two-sided Wald tests with Benjamini–Hochberg FDR correction for multiple testing. Shrunken log2 fold changes were estimated using lfcShrink(), and pairwise contrasts were used to compare life stages and treatments. Asterisks denote significance on a scale: padj <0.05 *, padj <0.005**, padj <0.0005 ***. Exact *p* values are (**A**) mysosin-regulating glycosidase, larvae *p* = *0.008*, pupae *p* = *0.003*; beta-glucuronidase, pupae *p* = *0.03*; beta-galactosidase, pupae *p* = *0.03*; glycoside hydrolase, pupae *p* = *0.009*; alpha glucosidase, pupae *p* = *0.003;* **B** sugar transport-like (1), pupae *p* = 0.01; sugar transport-like (2), pupae *p* = 0.006; sugar transport-like (3), pupae *p* = 0.0006; (**C**) trehalose-6-phosphate synthase, pupae *p* = 0.0007; facilitated trehalose transporter (1), pupae *p* = 0.03; facilitated trehalose transporter (2), pupae *p* = 0.009; facilitated trehalose transporter (3), pupae *p* = 0.003.

Functional enrichment of DEGs revealed host genes likely co-opted to support symbiont recovery just before pupation, when symbiont function is most critical. Most notably, genes

involved in sugar metabolism and transport were highly upregulated in symbiotic pupae compared to aposymbiotic individuals (Fig. 4A–B). This includes five genes that can break down sucrose, fructose, or lactose into β-D-galactose and β-D-glucose—substrates usable by *Westeberhardia—*coinciding with the rapid increase in symbiont titre (Fig. 4A). Several sugar transporters were also functionally enriched at this stage and more highly expressed in symbiotic pupae (Fig. 4B). These sugars could serve as an energy source by the symbiont but can also be fed directly into the shikimate pathway to produce 4-hydroxyphenylpyruvate, the molecular precursor that can be converted into tyrosine by the host (Fig. 4C). The sugar-metabolising gene myogenesis-regulating glycosidase was strongly expressed in symbiotic larval gut tissue (Fig. S4A–B). This suggests that hosts may increase carbohydrate intake during this life stage—similar to a mechanism in weevils that promotes endosymbiont proliferation^21^—then convert these into symbiont-compatible sugars before trafficking them to bacteriocytes.

Gene expression was visualised using RNA-FISH with hybridisation chain reaction (HCR): *Westeberhardia* was localised by tagging 16S ribosomal mRNA (yellow) using Alexa-514 fluorophore, and host trehalose-6-phosphate synthetase mRNA (magenta) using Alexa-647 fluorophore; host nuclei were stained with DAPI. Inset shows the same third-instar larva under brightfield with DAPI and RNA-FISH overlaid.

Our results also suggest symbiotic colonies may promote a more stable biochemical environment for *Westeberhardia* during pupation via trehalose-6-phosphate production and immune modulation. We find that trehalose-6-phosphate synthesis and transport genes were enriched in symbiotic pupae, and expression appears concentrated in bacteriocytes (Fig. 4D–E). In the *Burkholderia*–bean bug symbiosis, trehalose is produced by the host to support symbiont colonisation by providing stress protection and metabolic stability^22,23^. Although we cannot rule out that *Westeberhardia* is using trehalose as an energy source, it lacks the trehalase genes needed to cleave it, making the concentrated expression of trehalose transporters in bacteriocytes conspicuous. Instead, trehalose may help stabilise the symbiont’s environment during periods of rapid physiological change. In addition, we observed reduced expression of genes regulating potassium ion channel activity in symbiotic colonies (Fig. S5), which play a role in immune defence in honey bees^24,25^. Additionally, genes involved in MAPK signalling and apoptosis were more highly expressed in the absence of the symbiont (Fig. S5), suggesting that *C. obscurior* may actively suppress its immune response when *Westeberhardia* is present. Similar immune modulation has been reported in other insect symbioses—cereal weevils use IMD pathway mediated genes to prevent endosymbiont escape from bacteriocytes and, contrastingly, aphids have entirely lost the IMD pathway, possibly in response to tolerance of *Buchnera aphidicola*^26,27^. Together, these findings suggest that during metamorphosis, a period of major physiological change, the host may downregulate immunity and promote a stabilising biochemical environment to facilitate symbiont recovery and re-establishment in developing bacteriocytes.

### Hosts adaptively regulate metabolism to meet tyrosine demands

**Fig. 5:**
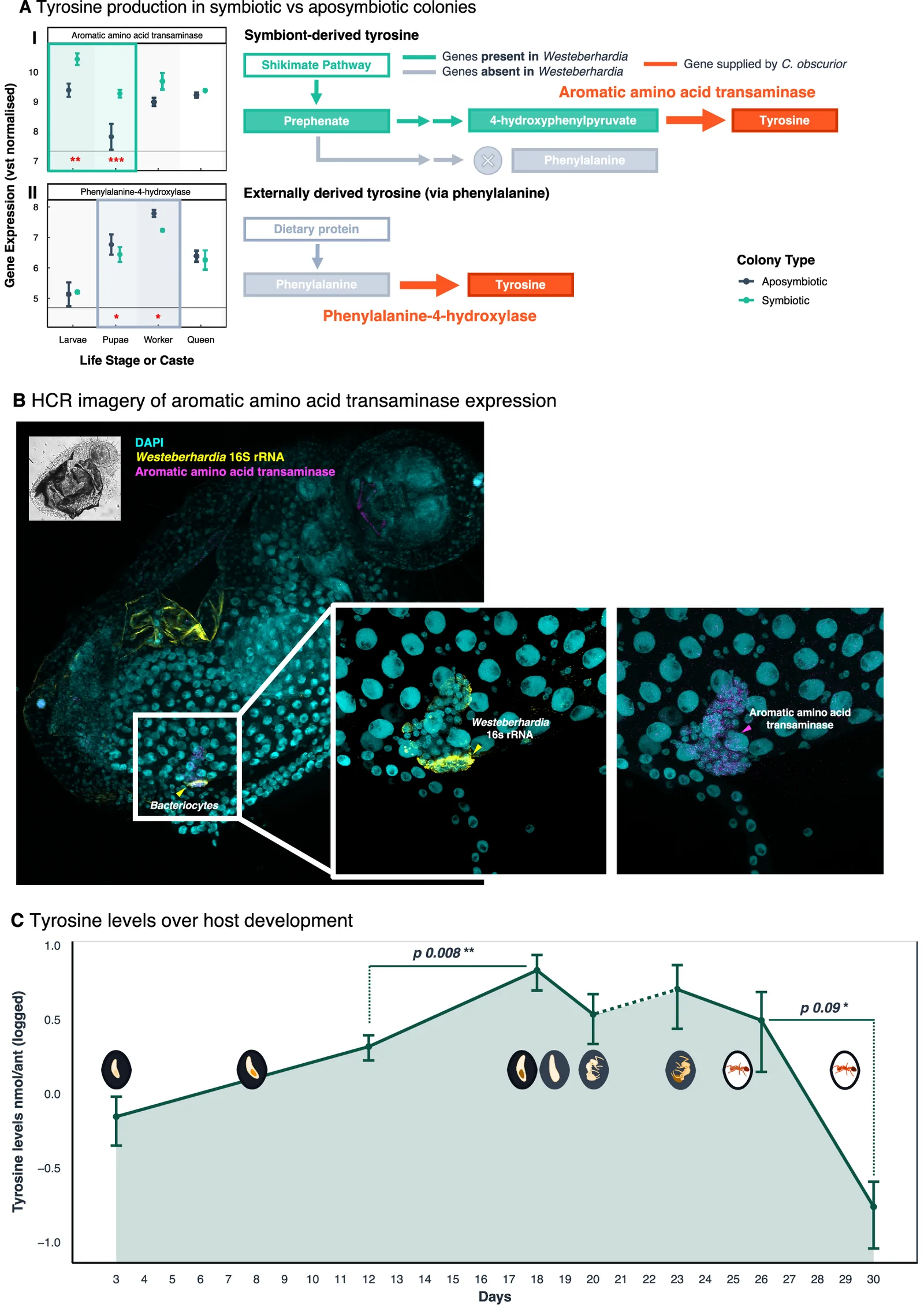
Flexible tyrosine biosynthesis strategies. **A** Expression of genes involved in two alternative pathways to produce tyrosine: (I) symbiont-derived 4-hydroxyphenylpyruvate converted by host aromatic amino acid transaminase (*tyrB*) (favoured in symbiotic larvae and pupae), and (II) host-derived conversion of dietary phenylalanine via phenylalanine-4-hydroxylase (*phhA*) (favoured in aposymbiotic pupae and workers); schematics indicate host vs. symbiont contribution (*n* = 3 biological replicates per life stage or caste, data are presented as mean values +/- SEM). Differential gene expression analysis was performed using DESeq2 in R with negative binomial generalised linear models. Statistical significance was assessed using two-sided Wald tests with Benjamini–Hochberg FDR correction for multiple testing. Shrunken log2 fold changes were estimated, and pairwise contrasts were used to compare life stages and treatments. Asterisks denote significance on a scale: padj <0.05 *, padj <0.005**, padj <0.0005 ***. Exact *p* values are: aromatic amino acid transaminase, larvae *p* = 0.008, *p* = 0.0001; phenylalanine-4-hydroxylase, pupae *p* = 0.04, worker *p* = 0.03; **B** *tyrB* expression localised to larval bacteriocytes using RNA-FISH via HCR (*Westeberhardia* 16S ribosomal mRNA in yellow; *C. obscurior tyrB* mRNA in magenta); **C** Tyrosine levels (nmol/ant) across development of symbiotic individuals; tyrosine is the only amino acid to increase during pupation (days 20–23, dotted line), when feeding has ceased. Differences between life stages were tested using linear regression analysis followed by Tukey-adjusted two-sided Wald *z* tests for pairwise comparisons, *n* = 9 per stage, data are presented as mean values +/- SEM (Fig. S6A).

Unlike many symbiotic insects with specialised diets, ants are omnivores and must flexibly manage nutrients from both symbiotic and exogenous sources. Remarkably, we find that *C. obscurior* meets this challenge by switching between two tyrosine biosynthesis pathways depending on whether the symbiont is present or not (Fig. 5A).

When *Westeberhardia* is present, we find that host and symbiont metabolism align to biosynthesise tyrosine: the symbiont supplies 4-hydroxyphenylpyruvate via the shikimate pathway, while the host completes the process using its aromatic amino acid transaminase (*tyrB*). We find that *tyrB* is strongly expressed during larval and pupal stages in symbiotic colonies (Fig. 5A). Expression is also localised to bacteriocytes, indicating that the host is actively converting symbiont-derived 4-hydroxyphenylpyruvate on-site into tyrosine ahead of cuticle formation (Fig. 5B). Our metabolomic analysis also revealed that tyrosine was the only amino acid to increase during pupation—a stage when feeding ceases and no new protein is being ingested (Fig. 5C and S6A) and to remain consistently elevated in symbiotic colonies under protein starvation (*F* = 6.6, *df* = 27, *p* < 0.02; Figure S6B, Supplementary Data 1). Additionally, expression of a cation transporter capable of shuttling L-DOPA, the tyrosine derivative needed for cuticle development, showed strong expression in bacteriocytes (Figure S4C).

In contrast, when the symbiont is absent, we find the host relies on phenylalanine-4-hydroxylase (*phhA*) to convert dietary phenylalanine into tyrosine—an alternative pathway more highly expressed in workers and pupae lacking the symbiont (Fig. 5A, II). This metabolic switch highlights the host’s flexibility in maintaining tyrosine supply in the absence of the symbiont and suggests that symbiont loss may be tolerable in protein-rich environments, potentially explaining the natural variability in *Westeberhardia*’s presence across host populations. Together, these results show that coordinated host–symbiont gene expression promotes tyrosine and cuticle precursor synthesis under nutrient limitation, while host metabolic flexibility ensures these essential pathways are maintained in the symbiont’s absence.

### Cardiocondyla exploits symbiont-derived folate provisioning when present

**Fig. 6:**
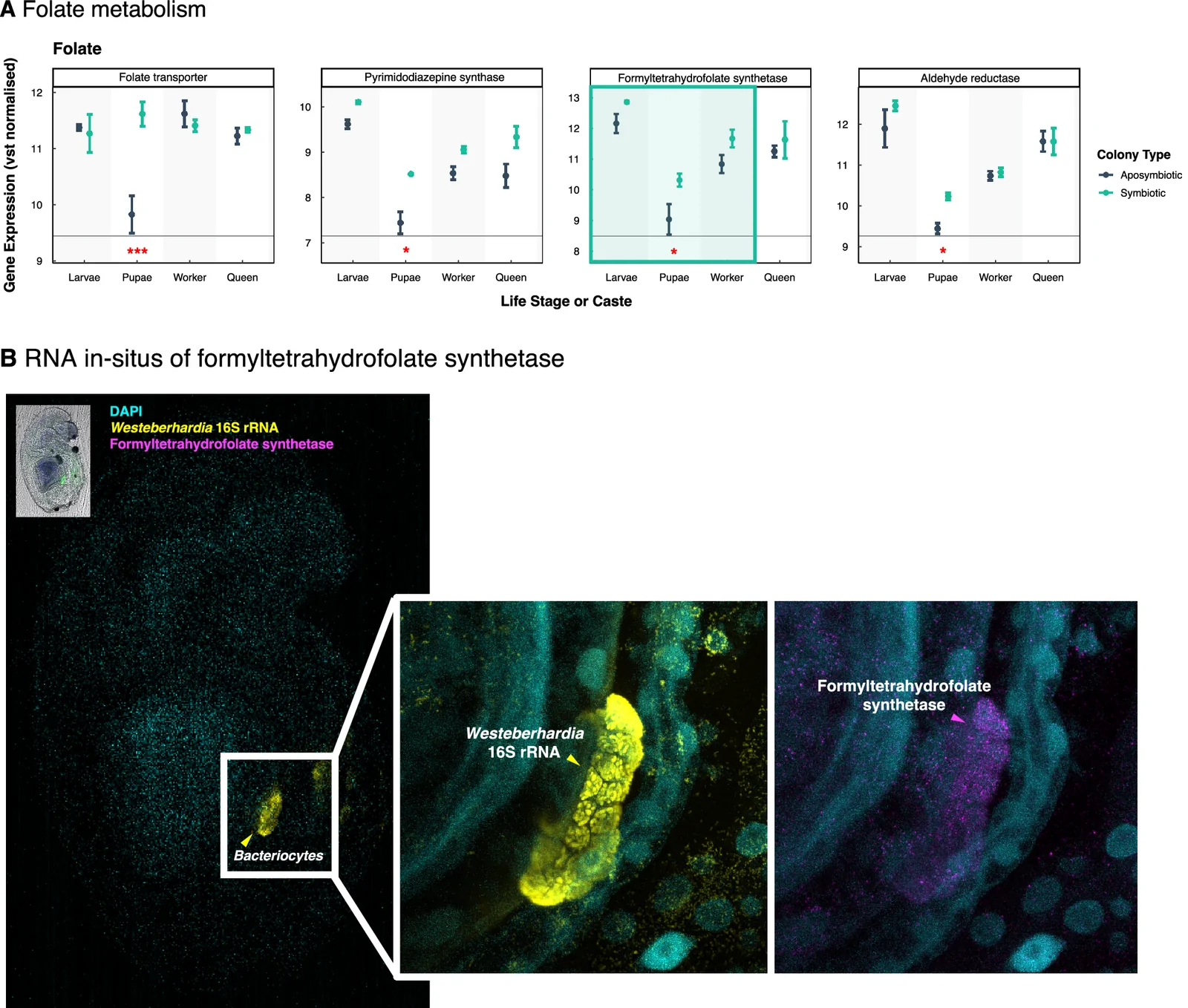
Folate provisioning in symbiotic colonies. **A** Host genes involved in folate metabolism, including formyltetrahydrofolate synthetase, a folate transporter and two genes involved in downstream processing, are more highly expressed in symbiotic colonies (*n* = 3 per life stage or caste). Differential gene expression analysis was performed using DESeq2 in R with negative binomial generalised linear models. Significance was assessed using two-sided Wald tests with Benjamini–Hochberg FDR correction for multiple testing. Shrunken log2 fold changes were estimated and pairwise contrasts were used to compare life stages and treatments. Asterisks denote significance on a scale: padj <0.05 *, padj <0.005**, padj <0.0005 ***. Exact *p* values are: folate transporter, pupae *p* = 0.000005; pyrimidodiazepine synthase, pupae *p* = 0.02; formyltetrahydrofolate synthetase, pupae *p* = 0.01; aldehyde reductase, pupae = 0.02; **B** Formyltetrahydrofolate synthetase is shown to be localised to larval bacteriocytes using RNA-FISH via HCR hybridisation (*Westeberhardia* 16S ribosomal mRNA in yellow green; *C. obscurior* formyltetrahydrofolate synthetase mRNA in magenta).

We also found that genes involved in folate (vitamin B_9_) metabolism and transport were significantly enriched in symbiotic colonies, suggesting symbiont-derived folate is important for host development. Notably, formyltetrahydrofolate synthetase is highly expressed in symbiotic pupae (Fig. 6A) and its expression is localised to larval bacteriocytes (Fig. 6B). This host enzyme is essential for carbon cycling and nucleotide synthesis, both critical for host growth and development. Folate provisioning by symbionts is common in blood-feeding insects^4,28–30^ and has also been documented in sap-feeding aphids^31^. *Westeberhardia* in *C. obscurior* has retained all genes for folate synthesis, however, some lineages of *Westeberhardia* have lost the capacity to produce folate, suggesting *Cardiocondyla* species may differ in their reliance on this as a symbiont-derived resource^13^.

## Discussion

### Environmentally responsive metabolism in insects

Metabolic plasticity is a key feature of insect adaptation, enabling responses to environmental cues, such as seasonality and diet, that have driven evolutionary innovations such as caste differentiation, diapause, and migration^32–36^. Our findings suggest that *Cardiocondyla obscurior* extends this plasticity to the regulation of its intracellular nutritional symbiont *Westeberhardia*. We show that ants can dynamically shift tyrosine biosynthesis between a symbiont-dependent route and a host-intrinsic pathway, allowing colonies to maintain fitness in the presence and absence of the symbiont.

Metabolic flexibility of this kind is likely rare in ancient intracellular symbioses, where hosts often become obligately reliant on symbiont-derived nutrients^6,37,38^. In contrast, *C. obscurior* has retained functional alternatives, activating symbiont-independent phenylalanine hydroxylation when *Westeberhardia* is absent. In most ancient nutritional symbioses, symbiont loss is accompanied by replacement that may result in some metabolic rewiring to accommodate the new symbiont^39^. True evolutionary losses of symbionts are relatively rare, but have occurred in hosts that transitioned to more nutrient-rich diets—scenarios that would also require the maintenance of pre-existing metabolic capacities to exploit these resources^9,40^. This study provides experimental evidence of a host mechanism that compensates for the loss of a strictly vertically transmitted nutritional symbiont, ensuring host survival. This metabolic versatility may reflect an evolutionary legacy of omnivory, which could favour the retention of redundant or alternative nutrient pathways. It raises the intriguing possibility that other ancient symbiotic omnivores, such as cockroaches^41^ and other ants^13^, may also preserve similar metabolic plasticity. However it may also be that the highly streamlined functional role of *Westeberhardia*, provisioning only shikimate-derived products, makes it more expendable in nature when compared to symbionts in omnivores with greater metabolic capacity—such as *Blochmannia* and *Blattabacterium*^41,42^.

### Host regulation and symbiont loss in nature

The facultative nature of *Westeberhardia* contrasts sharply with the widely held assumption that ancient, vertically transmitted nutritional endosymbionts are fixed and indispensable^13,37^. Our results support a model in which host control over symbiont density and function allows for conditional investment in the symbiosis, depending on nutritional context. Symbiont density increased under protein deprivation and during pupation. Transcriptomic data suggests that this regulation is predominantly mediated by the host, with no evidence of developmental stage-specific gene expression from the symbiont itself. Such host control may facilitate symbiont loss in environments where dietary protein is sufficient and may help explain why *Westeberhardia* is naturally absent in some *Cardiocondyla* queens and colonies^12,13^.

Our findings lend empirical support to the hypothesis that the dietary flexibility associated with omnivory may reduce dependence on symbiont-derived nutrients and promotes the persistence of labile symbioses. Importantly, we find that *C. obscurior* can not only tolerate symbiont loss but can also actively reconfigure its metabolism in response—a key distinction from other systems where symbiosis breakdown can lead to fitness costs or mortality, with functional replacement often documented^43–46^. Similar symbiont losses have been documented in other ant genera, such as the distantly-related *Formica*, where the symbiont’s presence varies across colonies, queens and species, despite long-term vertical transmission and bacteriocyte localisation^13^. The distribution of labile symbioses does not appear to be phylogenetically determined, as labile symbioses such as in *Formica* are closely related to stable ones, such as *Camponotus*. This suggests symbiont function and host ecology may be more important than shared ancestry in determining the stability of symbioses, at least in ants.

### Nutritional symbiosis in a social context

In social insects, colony nutrient demands drive selection on endosymbiont maintenance and regulation in colony members^13,47^. We find that *Westeberhardia* contributes to colony-level fitness by increasing brood survival and adult emergence under protein stress, and by enhancing worker size even under optimal conditions. Worker numbers are positively correlated with queen production in *C. obscurior*, therefore investment in workers under protein stress may increase colony fecundity^48^. Notably, we observed no difference in cuticle investment across colony types, despite the link between symbiont-derived tyrosine and cuticle strength in other insects^45,49^. Tyrosine is required in high quantities during cuticle biosynthesis^16^. Our results suggest symbiont-derived tyrosine is essential for exoskeleton development and adult eclosion in *C. obscurior*. We hypothesise that tyrosine may be the key limiting nutrient in arboreal habitats—where *C. obscurior* nests and forages—rapidly compromising colony fitness when in short supply^15,50^. This could explain why symbiont-derived tyrosine appears to support reproduction, worker size, and colony growth in *C. obscurior* rather than investment in cuticle thickness. Further tyrosine supplementation experiments could help clarify the extent of its contribution to rescuing protein-starved phenotypes.

Maintaining multi-queen colonies (polygyny) is often correlated with larger colony sizes and higher reproductive output^51^. Interestingly, all *Cardiocondyla* lineages that have undergone true evolutionary losses of *Westeberhardia* have transitioned to single-queen (monogynous) colonies, whereas symbiont-retaining lineages have retained their ancestral polygynous state^13^. Given that *Westeberhardia* enhances brood production and survival, the symbiont may also play a role in maintaining this energetically demanding social strategy, particularly under protein-limited conditions. Moreover, several *Cardiocondyla* lineages, including *C. obscurior*, have spread globally through the tropics and sub-tropics, and this invasive behaviour has only been observed in polygynous species^19^. The ability to modulate symbiont function in response to environmental conditions may buffer colonies against dietary variability, supporting traits commonly associated with invasion success, such as generalist diets and reproductive resilience^52–55^.

Our findings suggest that a context-dependent symbiosis contributes not only to nutritional homoeostasis but may also enhance this species’ broader ecological plasticity. We propose that a flexible nutritional symbiosis—coupled with the ability to modulate it—may enhance this plasticity, allowing *C. obscurior* and possibly other *Cardiocondyla* species to thrive under variable or suboptimal conditions encountered during colonisation.

## Methods

### Insect collection and maintenance

*Cardiocondyla obscurior* colonies used in this study were originally collected in Japan in 2011 by Jan Oettler and colleagues^12^ and reared under controlled conditions at the University of Regensburg. In June 2022, six of these colonies were transferred to Queen Mary University of London, where they were maintained in a climate-controlled incubator replicating Regensburg conditions: a 12:12 hr light:dark cycle with corresponding temperatures of 26 °C (day) and 22 °C (night), and 75% relative humidity. Colonies were fed three times per week with organic honey, cockroaches (one head or two legs provided once weekly), and fruit flies (five whole individuals provided twice weekly).

### Symbiont removal and validation

To remove *Westeberhardia*, *C. obscurior* colonies were heat-treated at 31 °C for four weeks, maintaining all other conditions constant (adapted from Fan & Wernegreen, 2013)^56^. To confirm symbiont removal, DNA was extracted from larvae using the Qiagen DNeasy Blood & Tissue kit (QIAGEN Ltd., Manchester, UK), and screened via diagnostic PCR targeting the 16S rRNA gene (primers: WeBh_F2-for: 5′-CATTTGAATATGTAGAATGGACC-3′; WeBh-R2: 5′-AACTTTTACAAGATCGCTTCTC-3′). PCR conditions were: 95 °C for 3 min; 39 cycles of 95 °C for 5 s, 60 °C for 20 s, 95 °C for 10 s; and a final elongation at 72 °C for 5 min. Amplicons were run on 10% agarose gels; only colonies with no visible band were considered aposymbiotic. Larvae were re-sampled regularly to confirm the aposymbiotic state. Aposymbiotic colonies were rested for four weeks and allowed to re-queen, achieved by retention of new alates and removal of old mated queens, before experimentation.

### RNA isolation, library prep, and sequencing

Ants were flash-frozen and stored at –80 °C. Samples from symbiotic and aposymbiotic colonies were pooled by life stage: (1) larval instars 1–3, (2) white and coloured pupae, (3) newly eclosed workers (identified by colour and mobility), and (4) queens. Samples within a treatment were pooled (*n* = 10) to obtain sufficient RNA and homogenise potential variation among individuals.

Total RNA was extracted from whole bodies using TriReagent® and the Zymo Direct-zol® Mini Kit following the manufacturers protocol. Samples were homogenised using pestles in 1.5 ml RNase-free Eppendorf tube with liquid Nitrogen. RNA integrity was verified via Agilent TapeStation, and only samples with RIN scores >8.5 were used for sequencing. Due to low RNA yield in some samples, both TruSeq® Stranded Total RNA Library Prep Kit (Illumina) and the SMARTer® Stranded Total RNA-Seq Kit v2 (Takara) were used. Ribosomal RNA was depleted using both Illumina RiboZero Gold and NEBNext® rRNA Depletion Kits on all samples to maximise efficiency. Illumina sequencing, rRNA depletion and library preparation was performed by Macrogen (UK), generating ≥60 million 100 bp paired-end reads per sample. Sample details can be found Supplementary Data 2.

### Gene expression analysis

Reads were trimmed of the first 5 bases, adaptors, and those with PHRED scores ≥25 using Trim Galore^57^, then checked with FastQC and MultiQC^58,59^. Residual rRNA was removed using SortMeRNA^60^. Reads were mapped to concatenated host and symbiont genomes using STAR aligner to maximise mapping fidelity^12,13,61,62^.

Raw STAR counts were analysed in R using DESeq2 (for differential gene expression) and limma (for batch correction via variance stabilising transformation)^63,64^. Pairwise comparisons between symbiotic and aposymbiotic life stages were conducted using lfcshrink, with significance defined as adjusted p < 0.05. Visualisations used ggplot2^65^. Genes of interest were identified through enrichment analyses of DEGs. To maximise detection of enriched genes, including poorly annotated but potentially important ones, we used multiple annotation tools—INTERPRO, Panther, GO, and KEGG—in combination with the universal enrichment function from the clusterProfiler^66–70^. Enriched genes and pathways were manually verified and further annotated using NCBI’s BLAST^71^. Full gene expression data and annotations are in Supplementary Data 3–5; code is in Supplementary Information.

### Gene expression localisation using hybridisation chain reaction

RNA fluorescent in situ hybridisations (RNA-FISH) using hybridisation chain reaction (HCR) were used to validate patterns of spatial gene expression (adapted from Ferreira et al.)^72^. Whole larvae were fixed in 37% paraformaldehyde/PBS/heptane (1:1:2) for 2-h, then hybridised with gene-specific oPools Oligo Pools probes (from IDT, sequences reported in Supplementary Data 14 in Supplementary Information) for 36–48 h in a water bath at 37 °C. Amplifier hairpins (Molecular Instruments) were applied overnight in the dark using a rocking table^73,74^, followed by staining of host tissue using DAPI diluted in PBS (10X) at a ratio of 1:1000 for 3 h. Probes targeting *Westeberhardia* 16S rRNA (Alexa-514) were used to locate bacteriocytes; host genes were labelled with Alexa-647. Whole larvae were mounted on slides using glycerol and gene frames (Fisher Scientific, Leicestershire, UK, LE11 5RG). Imaging was conducted on a Leica Stellaris 8 confocal microscope and processed using Fiji (also known as ImageJ)^75^. Full HCR protocol is in the Supplementary Information.

### Diet manipulations and phenotypic analysis

To simulate dietary protein limitations, colonies were assigned to one of three diets: (1) control (insects and honey 3×/week), (2) low-protein diet (insects 1×/4 weeks, honey 3x/week), and (3) no-protein (honey only 3×/week). In weeks when low-protein colonies received insects, they were given the same number as control colonies, so only the frequency of protein provisioning differed. Prior to diet assays, each symbiotic colony (derived from seven separate source colonies) was split into two; one half was heat-treated to remove the symbiont (Methods 1.2). After a four-week recovery, both symbiotic and now aposymbiotic colonies were each divided into three sub-colonies, followed by an additional two-week rest. Aposymbiotic status was regularly confirmed by PCR (Methods 1.2, Supplementary Information). Prior to treatment, all colonies were standardised with two mated queens and adjusted brood levels. Dead individuals were removed daily to reduce cannibalism risk. All colonies were handled identically.

### Fitness assays

To assess pupae size, individuals were photographed blind and measured using a calibrated graticule. Pupal size differences were assessed with Welch’s *t* tests^76^. Following dietary treatment initiation, brood production was recorded three times weekly using non-invasive stereomicroscopy. Every three weeks, samples were collected for: (1) cuticle analysis (newly eclosed workers), (2) metabolomics (late pupae), and (3) symbiont titre (third-instar larvae). Colony mortality was recorded throughout the experiment and analysed using Cox proportional hazards models with colony identity included as a random effect. For analysis of brood production, we used generalised linear mixed effects modelling (glmer.nb from package lme4 in R), with a non-binomial distribution to compensate for overdispersion, and colony identity as a random effect^77^. All models tested for effects of symbiotic status (symbiotic vs. aposymbiotic), diet (control, low protein, no protein), and their interaction over time. Models were compared using likelihood ratio tests (LRT) and Akaike’s Information Criterion (AIC) and the R package emmeans was used to assess pairwise comparisons^78^. Visualisations were generated using *ggplot2*^65^. Fitness assay data is available in Supplementary Data 6 and statistical model outputs in Supplementary Data 1.

### Symbiont density

Third-instar larvae (~day 13) were flash-frozen and used for DNA extraction (Qiagen DNeasy kit). qPCR was performed using primers for *Westeberhardia* gene nrdB (ribonucleoside-diphosphate reductase 1 subunit beta)—5’ – GGA AGG AGT CCT AAT GTT GCG – 3’ and 5’ – AGC GTG CTC ACG AGT TTG TCC G – 3’—and host gene EF-1 (elongation factor 1-alpha 1)— 5’ –TCA CTG GTA CCT CGC AAG CCG A– 3’ and 5’ – ACC AGA AAT ATC TTT TGC ACG TT – 3’^12^. Each 10 µL reaction contained Lunar universal mix (NEB), 2 µL template, and 0.1 µL of each primer. Conditions: 95 °C for 3 min; 39 cycles; melt curve 65–95 °C in 0.5 °C increments. qPCRs were run on 202 individuals in the same week with a single standard curve (values between 90–110% were accepted) (Supplementary Data 7). Data were analysed with linear models (lme4) in R^77^, testing the difference in titre between colonies on different diets treatments at specific time points (week 0, week 6 and week 12). Third instar larvae were selected for symbiont titre measurements because they showed the lowest variation compared to other developmental stages. This reduced the likelihood that natural variation would obscure overall trends and increased our ability to detect treatment effects. Results are in Supplementary Data 1.

### Metabolomics

To measure amino acid levels, late-stage pupae were frozen, crushed under liquid nitrogen, and stored in 500 µL of 80% HPLC-grade methanol before being sent to the Max Planck Institute in Jena, Germany, for processing. There was no separation of crushed material from the supernatant as the ant material was extremely small. Two negative controls were included. Due to sample limitations, amino acids were measured only in the control and low-protein diet treatments. Each 50 µL sample was diluted with 50 µL of an aqueous mix of ^13^C,^15^N-labelled amino acid standards (Aldrich, cat. no. 487910). Late-stage pupae were intentionally selected as this is when the cuticle begins to melanise and sclerotise, and so we would expect the presence and metabolism of tyrosine at this stage.

Samples were kept at 10 °C, and 1 µL was injected into an UltiMate™ 3000RS UHPLC system with a Zorbax Eclipse XDB-C18 (3.0 × 50 mm, 1.8 µm) column, using acidified water and methanol in gradient mode^79^ (Schäfer et al., 2016). Natural amino acid isomers and internal standards were detected via EVOQ™ Elite triple-quadrupole MS (Bruker) with heated electrospray ionisation in positive mode (Spray voltage: 4500 V; Cone gas: 35 AU; Cone temp: 350 °C; Probe gas: 50 AU; Probe temp: 500 °C; Nebuliser gas: 60 AU). Samples were analysed in MRM mode, and concentrations were calculated using internal standards as per Schäfer et al.^79^. Post-run analysis was done using Bruker MS Workstation (v8.2.1). Generalised linear mixed effects models were used to test amino acid differences between colony types across diets, with colony identity as a random effect. Further methodological details can be found in Supplementary Information, along with full calculations and concentrations in Supplementary Data 8 and 9; model results are in Supplementary Data 1.

### Cuticle thickness

Newly emerged workers were fixed in 4% formaldehyde in PBS, stored in ethanol, and sent to the Max Planck Institute (Jena, Germany). For micro-CT analysis, ants were fixed for 24 h in a 1:2 mix of 40% paraformaldehyde in PBS and 80% ethanol (Carl-Roth), washed in 100% ethanol, and then critical point dried (Leica EM CPD3000; 35 CO₂ cycles, low speed, 40 °C). Samples were mounted and scanned using a Skyscan 1272 (Bruker; HAMAMATSU L10101-67 X-ray source, XIMEA xiRAY16 camera) at 1.000023 µm resolution, 0.105° rotation steps, 38 kV, 130 µA, 4× image averaging, 2 × 2 binning, 546 ms exposure.

3D datasets were reconstructed with NRecon (v2.1.0.1) using smoothing (1 iteration, kernel 2), ring artefact (0 or 5), and beam hardening correction (0–20%). Analysis in Dragonfly (v2022.1.0.1259, ORS) involved segmentation via upper OTSU threshold, followed by automated (close, fill X/Y/Z, erode, smooth; kernel ≤11) and manual refinement to isolate cuticle. Body and cuticle volumes were used to calculate proportional cuticular investment. Cuticle thickness meshes were generated with Laplacian smoothing (3 rounds) to extract average, median, and SD of thickness across the full body using Dragonfly. Full data are in Supplementary Data 10.

### Reporting summary

Further information on research design is available in the Nature Portfolio Reporting Summary linked to this article.

## Supplementary information

Supplementary information

Descriptions of Additional Supplementary Files

Supplementary Data 1

Supplementary Data 2

Supplementary Data 3

Supplementary Data 4

Supplementary Data 5

Supplementary Data 6

Supplementary Data 7

Supplementary Data 8

Supplementary Data 9

Supplementary Data 10

Supplementary Data 11

Supplementary Data 12

Supplementary Data 13

Supplementary Data 14

Reporting Summary

Transparent Peer Review file

## Data Availability

The raw sequencing data generated in this study have been deposited on NCBI^80^ (BioProject ID PRJNA1344296, BioSamples accessions SAMN52633624: SAMN52633647) and can be accessed at https://www.ncbi.nlm.nih.gov/bioproject/PRJNA1344296/ (https://www.ncbi.nlm.nih.gov/bioproject/PRJNA1344296/). The sample metadata files are available in figshare^81^: (https://doi.org/10.6084/m9.figshare.30608795) Metabolomics data (raw data as well as an associated method description and raw quantification results) are deposited in Edmond, the community data repository of the Max-Planck-Society, (https://doi.org/10.17617/3.MLVYYE [https://doi.org/10.17617/3.MLVYYE)^82^.

## Code availability

Code associated with this analysis can be found on figshare^81^: https://doi.org/10.6084/m9.figshare.30608795 (https://doi.org/10.6084/m9.figshare.30608795).
