# Supplementary material for: Metabolic plasticity supports a flexible nutritional symbiosis in Cardiocondyla ants
Source: Nat Commun. 2026 Aug 21;17:10002. doi: 10.1038/s41467-026-76801-z (PMC13590504; doi:10.1038/s41467-026-76801-z)
Supplement: Supplementary file 1 [file 41467_2026_76801_MOESM17_ESM.pdf]

nature portfolio

Corresponding author(s): Lee Henry

Last updated by author(s): 15/05/26

Reporting Summary

Nature Portfolio wishes to improve the reproducibility of the work that we publish. This form provides structure for consistency and transparency in reporting. For further information on Nature Portfolio policies, see our Editorial Policies and the Editorial Policy Checklist.

Statistics

For all statistical analyses, confirm that the following items are present in the figure legend, table legend, main text, or Methods section.

n/a	Confirmed	
☐	☒	The exact sample size (*n*) for each experimental group/condition, given as a discrete number and unit of measurement
☐	☒	A statement on whether measurements were taken from distinct samples or whether the same sample was measured repeatedly
☐	☒	The statistical test(s) used AND whether they are one- or two-sided<br>*Only common tests should be described solely by name; describe more complex techniques in the Methods section.*
☐	☒	A description of all covariates tested
☐	☒	A description of any assumptions or corrections, such as tests of normality and adjustment for multiple comparisons
☐	☒	A full description of the statistical parameters including central tendency (e.g. means) or other basic estimates (e.g. regression coefficient) AND variation (e.g. standard deviation) or associated estimates of uncertainty (e.g. confidence intervals)
☐	☒	For null hypothesis testing, the test statistic (e.g. *F*, *t*, *r*) with confidence intervals, effect sizes, degrees of freedom and *P* value noted<br>*Give P values as exact values whenever suitable.*
☒	☐	For Bayesian analysis, information on the choice of priors and Markov chain Monte Carlo settings
☐	☒	For hierarchical and complex designs, identification of the appropriate level for tests and full reporting of outcomes
☒	☐	Estimates of effect sizes (e.g. Cohen's *d*, Pearson's *r*), indicating how they were calculated

Our web collection on statistics for biologists contains articles on many of the points above.

Software and code

Policy information about availability of computer code

Data collection	No software was used for data collection.
Data analysis	Existing software tools such as "trimmomatic" and "STAR aligner" were used for data cleaning with custom scripts built for our sequences. Statistical analysis and visualization was performed using existing R packages such as "lme4" and "ggplot2". No custom packages or software tools were use for data analysis.

For manuscripts utilizing custom algorithms or software that are central to the research but not yet described in published literature, software must be made available to editors and reviewers. We strongly encourage code deposition in a community repository (e.g. GitHub). See the Nature Portfolio guidelines for submitting code & software for further information.

Data

Policy information about availability of data

All manuscripts must include a data availability statement. This statement should provide the following information, where applicable:

- Accession codes, unique identifiers, or web links for publicly available datasets
- A description of any restrictions on data availability
- For clinical datasets or third party data, please ensure that the statement adheres to our policy

All sequencing data associated with this research are available on NCBI (BioProject ID PRJNA1344296, BioSamples accessions SAMN52633624: SAMN52633647). All metabolomic data are available on Edmond, doi: https://doi.org/10.17617/3.MLVYYE. There are no restrictions on data availability.

Research involving human participants, their data, or biological material

Policy information about studies with human participants or human data. See also policy information about sex, gender (identity/presentation), and sexual orientation and race, ethnicity and racism.

Reporting on sex and gender	n/a
Reporting on race, ethnicity, or other socially relevant groupings	n/a
Population characteristics	n/a
Recruitment	n/a
Ethics oversight	n/a

Note that full information on the approval of the study protocol must also be provided in the manuscript.

Field-specific reporting

Please select the one below that is the best fit for your research. If you are not sure, read the appropriate sections before making your selection.

☐ Life sciences ☐ Behavioural & social sciences ☒ Ecological, evolutionary & environmental sciences

For a reference copy of the document with all sections, see nature.com/documents/nr-reporting-summary-flat.pdf

Ecological, evolutionary & environmental sciences study design

All studies must disclose on these points even when the disclosure is negative.

Study description	This study aims to characterize the mechanism of host control over intracellular bacterial symbionts and validate their functional role in Cardiocondyla ants. The study uses live ant colonies, reared under standard laboratory conditions. The species used harbors an intracellular bacterial symbiont which was removed from half the experimental groups and then the two groups (N=7 per group) were subjected to varying levels of protein while keeping all other conditions the same. Various colony fitness metrics were recorded (brood production, mortality) and modeled as response variables in a nested design modeling the effects of symbiotic status (symbiotic vs. aposymbiotic), diet (control, low protein, no protein), and their interaction over time. Metabolite data (amino acid levels) and symbiont density were also measured throughout the diet treatment and modeled in the same way. Worker size was measured after photographing the pupae blind between groups and tested using a simple t test. Additionally RNA sequencing data was obtained and analyzed for differentially expressed genes (DESeq2 in R) between colony types (symbiotic vs. aposymbiotic) at different life stages and castes —this data was then used to inform an RNA-FISH approach to detect gene expression localization in larvae harboring the symbiont.
Research sample	Cardiocondyla obscurior ants were the test species for this entire study. They are part of the family Myrmicinae, and known colloquially as tramp ants, with a reputation of invasiveness. The colonies were collected in Japan in 2011 and have been reared under standard laboratory conditions since collection. Cardiocondyla obscurior was chosen as they possess an ancient intracellular bacterial symbiont that is sometimes found to be absent in natural populations and can be gently removed using heat treatment in laboratory conditions. Symbiont-free colonies can be kept alive and healthy under the same standard lab conditions as symbiont-positive colonies, making them ideal for comparisons between symbiotic and aposymbiotic colonies.
Sampling strategy	For the phenotypic work (diet manipulations), we used N=7 colonies per treatment group, with extensive sub sampling of individuals within each colony. This level of replication was found to be sufficient to capture biologically meaningful variation at the colony level. For the RNA-sequencing analysis, we used N = 3 biological replicates per life stage/caste and treatment group. This sample size is standard and widely accepted for RNA-seq studies when accompanied by independent validation approaches, which we included in the form of RNA-FISH imaging and additional downstream analyses, thereby strengthening the robustness and reproducibility of the gene expression findings. For symbiont density testing, a minimum replicate of N=9 and maximum of N=16 was used to account for variation in symbiont density between individuals and colonies. N=13 (1-2pupae per colony) was used for pupae size measurements and samples were photographed and measured blind to avoid any bias. For metabolite sampling over life cycle, N=9 was used.
Data collection	In the phenotypic experiments (diet manipulations) data was recorded in a physical lab book during microscope observations and transfered to excel spreadsheets each week by PC. For RNA sequencing and symbiont density testing, samples were collected and processed in the lab at Queen Mary University London by PC, this data was cleaned, analyzed and visualized by same person. The metabolite data was collected by PC, and then processed by RH and TE. All RNA-FISH samples were collected and processed by PC. Worker pupae were collected and photographed blind by PC and a lab technician for worker pupae size measurements.
Timing and spatial scale	Diets treatments were conducted over a 12 week period. Aposymbiotic colonies were subjected to 4 weeks of heat treatment to remove the symbionts and then rested for 4 weeks to re-queen prior to the diet manipulations. During the 12 weeks, count data was collected 3 times per week (Monday, Wednesday and Friday). Collection of physical samples for metabolite measurements, cuticle thickness and symbiont density was attempted once every 4 weeks, resulting in 5 key time points (week 0, week 3, week 6, week 9,

	week 12). For RNA sequencing, final samples were collected and RNA extracted randomly over a 4 week period.
Data exclusions	No data was excluded from the sampling described above.
Reproducibility	Adequate replication for each element of the study was used to ensure reproducibility. For data analysis, several statistical approaches were tested before the final model was selected, and the final models were run several times to ensure agreement. All data and code will be made fully available to repeat the analysis.
Randomization	For RNA extractions, DNA extractions and PCR's (symbiont density), samples were randomly assigned extraction numbers to avoid batch effects or bias of extraction. For worker pupae measurement, pupae were selected, photographed and measured without prior knowledge of the colony type, again using a randomly allocated numbering system. For the phenotypic, metabolite and cuticle thickness data, randomization was not possible during the physical experiment, but random effects were included for colony identity when modeling the data. All extractions and sample processing were performed after the 12-week diet experiments were completed.
Blinding	Blinding was used when selecting, photographing and measuring the worker pupae for size comparison. Blinding was also used during molecular lab work such as RNA/DNA extractions and PCR reactions, where samples were numbered to hide their identity. Metabolite work was inherently blind as the facility carrying out the laboratory protocols were unaware of the naming convention of the samples.

Did the study involve field work? ☐ Yes ☒ No

Reporting for specific materials, systems and methods

We require information from authors about some types of materials, experimental systems and methods used in many studies. Here, indicate whether each material, system or method listed is relevant to your study. If you are not sure if a list item applies to your research, read the appropriate section before selecting a response.

Materials & experimental systems

n/a	Involved in the study	
☒	☐	Antibodies
☒	☐	Eukaryotic cell lines
☒	☐	Palaeontology and archaeology
☐	☒	Animals and other organisms
☒	☐	Clinical data
☒	☐	Dual use research of concern
☒	☐	Plants

Methods

n/a	Involved in the study	
☒	☐	ChIP-seq
☒	☐	Flow cytometry
☒	☐	MRI-based neuroimaging

Animals and other research organisms

Policy information about studies involving animals; ARRIVE guidelines recommended for reporting animal research, and Sex and Gender in Research

Laboratory animals	Cardiocondyla obscurior ant colonies.
Wild animals	n/a
Reporting on sex	n/a
Field-collected samples	n/a
Ethics oversight	No ethical approval is required for these invertebrates.

Note that full information on the approval of the study protocol must also be provided in the manuscript.

Plants

Seed stocks	n/a
Novel plant genotypes	n/a
Authentication	n/a
