# Supplementary material for: Metabolic plasticity supports a flexible nutritional symbiosis in Cardiocondyla ants
Source: Nat Commun. 2026 Aug 21;17:10002. doi: 10.1038/s41467-026-76801-z (PMC13590504; doi:10.1038/s41467-026-76801-z)
Supplement: Supplementary file 2 [file 41467_2026_76801_MOESM18_ESM.pdf]

Peer Review File

Metabolic plasticity supports a flexible nutritional symbiosis in Cardiocondyla ants

Corresponding Author: Dr Lee Henry

This file contains all reviewer reports in order by version, followed by all author rebuttals in order by version.

Version 0:

Reviewer comments:

Reviewer #1

(Remarks to the Author)
In this manuscript, Cunningham et al investigate host/symbiont interactions between the ant species Cardiocondyla obscurior and the bacterium Westeberhardia cardiocondylae. Specifically, the authors shed light on metabolic plasticity that supports the transient nature of the symbiotic pair observed in the wild. The authors demonstrate that colonization by W. cardiocondylae supports colony fitness during protein starvation. Their data support a model where, at pupation, the host upregulates sugar metabolism and uptake genes to funnel these substrates to the symbiont in exchange for precursors of tyrosine, a limiting resource that is critical for pupation. Importantly, in aposymbiont colonies, C. obscurior upregulates genes involved in scavenging tyrosine from phenylalanine under protein-starvation conditions, compensating for the lack of W. cardiocondylae.

General Comments
1) In line 318 of the discussion, the authors mention that other ant genera demonstrate transient relationships with their symbionts. It might help to have a bit more detail on the phylogenetic distribution of the these ant genera relative to ant genera where symbionts have not been found or are essential for survival. Do these genera with transient symbionts cluster?
2) The evidence is compelling that W. cardiocondylae boosts colony fitness during protein-starvation conditions. I'm curious though if tyrosine is the only factor at play. Did you perform any experiments where you feed the protein-starved aposymbiont ants sugar spiked with tyrosine to see how this affects colony fitness? Such an experiment would further inform on this symbiosis.
3) At line 191-192, you mention that the trehalose transporter gene is upregulated and localized to the bacteriocytes; however, in the full image you can see this transporter gene present at similar levels across other cells beyond the bacteriocytes. Is this really "localized" if it spans across all cells?
4) Is it possible that the more than 10 years of lab rearing before this study might have had an impact on the findings? For example, is it possible that the ant lost other symbionts that could play a role in the field?

Minor:
Line 80: fix 'hypothesise' and remove comma after 'that'
Line 217: fix 'biosynthesise'
Line 345: fix '…have are…'

Reviewer #2

(Remarks to the Author)
This manuscript presents a comprehensive multi-omics study examining how a natural symbiont influences ants' tolerance to protein scarcity. The work is substantial and the system is well suited to the questions posed. The findings suggest that ants rely on distinct pathways for tyrosine acquisition from symbiont-derived versus dietary sources, and that symbiont presence enhances tolerance to low-protein conditions. Detailed comments are provided below.

Major comments:
I'm not convinced that the observed changes in symbiont density across host development or under different diets can be

described as “actively regulated” by the host. The authors should be cautious when interpreting correlations. A higher symbiont load during cuticle-formation stages does not necessarily indicate a “host-regulated mechanism that prioritises symbiont recovery”. This phrasing implies that the host has full control over increasing or eliminating the symbiont, which is not supported by the current data.
Why were metabolomics performed only on late pupae, and symbiont titre measured only in third-instar larvae? It would strengthen the manuscript if the authors could justify these choices, rather than a collection of techniques applied to different samples without a connected story.
Line 385: “Aposymbiotic colonies were … allowed to re-queen before experimentation” – were they de-queened during the heat-treatment to remove Westeberhardia? And were the aposymbiotic colonies introduced with only one queen instead of multiple queens as the natural aposymbiotic colony?
Lines 442-444: What were the insects being fed here? Earlier it was mentioned the standard colonies were fed cockroaches (once weekly), and fruit flies (twice weekly). Were a combination of cockroaches and fruit flies being used in control and low protein diet? Please also specify the sources of the insects provided, and how they were fed – were whole fresh insects offered, or were they cut open, etc.?
Lines 444-445: “each symbiotic colony was split in two” - How many origin symbiotic colonies were used in this experiment? Were all the 6 groups (3 diet, 2 symbiotic status) derived from a single original colony, or were multiple colonies included?
Sample size is missing in some experiments, e.g., metabolomics.

Minor comments:
The abstract does not mention methods. It would be helpful to include the techniques used in the study to clarify the scope.
Line 31: spell out “Cand.” as it was first time mentioned.
Line 79: change to carbohydrate-to-protein
Lines 103-109: some “p” were italicized, some were not
Lines 126-132: add in statistical support
Figure 3: Since each bar represents upregulated transcripts within the comparison, it would be helpful to stack the dark blue and light blue pairs together for each caste or life stage (i.e., upregulated and downregulated side-by-side).
Line 338 and 358: Change the dash to emdash
Lines 406: What is the read length?

Version 1:

Reviewer comments:

Reviewer #1

(Remarks to the Author)
The authors have addressed my comments.

Reviewer #2

(Remarks to the Author)
The authors have addressed the comments sufficiently.

Open Access This Peer Review File is licensed under a Creative Commons Attribution 4.0 International License, which permits use, sharing, adaptation, distribution and reproduction in any medium or format, as long as you give appropriate credit to the original author(s) and the source, provide a link to the Creative Commons license, and indicate if changes were made.
In cases where reviewers are anonymous, credit should be given to 'Anonymous Referee' and the source.
The images or other third party material in this Peer Review File are included in the article's Creative Commons license, unless indicated otherwise in a credit line to the material. If material is not included in the article's Creative Commons license and your intended use is not permitted by statutory regulation or exceeds the permitted use, you will need to obtain permission directly from the copyright holder.
To view a copy of this license, visit https://creativecommons.org/licenses/by/4.0/

Manuscript number: NCOMMS-25-83864

Title: Metabolic plasticity supports a flexible nutritional symbiosis in *Cardiocondyla* ants

Authors: Phoebe Cunningham[1], José M Martín-Durán[1], Jan Oettler[2], Eva Shultner[2], Rayko Halitschke[3], Tobias Engl[4], Martin Kaltenpoth[4], Lee Henry[1*]

Overview response,

We thank the reviewers and editor for their comments and helpful suggestions. We have accepted all the points raised and implemented the reviewer's recommendations, with minor exceptions outlined below. Please find a detailed point-by-point response (in blue font) to all the comments and recommendations (in italics).

Specifically, we have:

- Tempered some of the wording around host regulation of symbiont titre to convey the message that fluctuations in symbiont titre may be host-assisted rather than actively host-controlled.
- Revised phrasing around localisation of trehalose transporter gene expression to better reflect observed patterns.
- Provided additional methodological details around the insect cultivation, life stage choices and diet manipulation experiments.
- Added statistical support to the text and supplementary material for life stage variation in symbiont titre.
- Addressed and corrected all minor comments.

REVIEWER COMMENTS

Reviewer #1 (Remarks to the Author):

In this manuscript, Cunningham et al investigate host/symbiont interactions between the ant species Cardiocondyla obscurior and the bacterium Westeberhardia cardiocondylae. Specifically, the authors shed light on metabolic plasticity that supports the transient nature of the symbiotic pair observed in the wild. The authors demonstrate that colonization by W. cardiocondylae supports colony fitness during protein starvation. Their data support a model where, at pupation, the host upregulates sugar metabolism and uptake genes to funnel these substrates to the symbiont in exchange for precursors of tyrosine, a limiting resource that is critical for pupation. Importantly, in aposymbiont colonies, C. obscurior upregulates genes involved in scavenging tyrosine from phenylalanine under protein-starvation conditions, compensating for the lack of W. cardiocondylae.

General Comments
1) In line 318 of the discussion, the authors mention that other ant genera demonstrate transient

relationships with their symbionts. It might help to have a bit more detail on the phylogenetic distribution of the these ant genera relative to ant genera where symbionts have not been found or are essential for survival. Do these genera with transient symbionts cluster?

This is an interesting point that we have now highlighted in the text. There is no phylogenetic clustering of ant hosts harbouring transient symbionts, and in fact we see lineages with labile symbiosis more closely related to lineages with stable symbiosis, such as *Formica* (labile) and *Camponotus* (stable). This suggests the symbiosis may provide different functions, with different degrees of necessity for the host, between labile and stable systems. We have now explicitly mentioned this in the discussion on lines 326-332.

2) The evidence is compelling that W. cardiocondylae boosts colony fitness during protein-starvation conditions. I'm curious though if tyrosine is the only factor at play. Did you perform any experiments where you feed the protein-starved aposymbiont ants sugar spiked with tyrosine to see how this affects colony fitness? Such an experiment would further inform on this symbiosis.

We agree that the tyrosine supplementation experiment proposed by the reviewer would be informative. However, resolving the precise metabolites underlying this effect would involve substantial additional experiments that are beyond the scope of the present study. Our aim was to demonstrate that *W. cardiocondylae* enhances colony performance under protein limitation and to investigate host regulation of this interaction, which our data supports independent of the specific metabolite involved. We have now explicitly highlighted this as an important future direction in the Discussion, lines 348–350.

3) At line 191-192, you mention that the trehalose transporter gene is upregulated and localized to the bacteriocytes; however, in the full image you can see this transporter gene present at similar levels across other cells beyond the bacteriocytes. Is this really "localized" if it spans across all cells?

We agree that "localized" was too strong of an assertion and have revised this to state that expression of the transporter appears concentrated in bacteriocytes. As trehalose is the principal haemolymph sugar in most insects, expression across multiple host cell types is expected. What is interesting about trehalose in this system is that it cannot be directly taken up by the symbionts, therefore the concentrated expression of trehalose transports into the bacteriocytes is conspicuous. We have now updated the text to reflect this nuanced aspect of the biology (line 192-197).

4) Is it possible that the more than 10 years of lab rearing before this study might have had an impact on the findings? For example, is it possible that the ant lost other symbionts that could play a role in the field?

Previous work by Jackson et al. (*ISME Journal*, 2022) compared natural and long-term laboratory populations, including those used in our study, and found no differences in symbiont

composition. This indicates that extended lab rearing did not alter the symbiont community.

Minor:
Line 80: fix 'hypothesise' and remove comma after 'that'
Fixed

Line 217: fix 'biosynthesise'
Fixed

Line 345: fix '…have are…'
Fixed

Reviewer #2 (Remarks to the Author):

This manuscript presents a comprehensive multi-omics study examining how a natural symbiont influences ants' tolerance to protein scarcity. The work is substantial and the system is well suited to the questions posed. The findings suggest that ants rely on distinct pathways for tyrosine acquisition from symbiont-derived versus dietary sources, and that symbiont presence enhances tolerance to low-protein conditions. Detailed comments are provided below.

Major comments:
I'm not convinced that the observed changes in symbiont density across host development or under different diets can be described as "actively regulated" by the host. The authors should be cautious when interpreting correlations. A higher symbiont load during cuticle-formation stages does not necessarily indicate a "host-regulated mechanism that prioritises symbiont recovery". This phrasing implies that the host has full control over increasing or eliminating the symbiont, which is not supported by the current data.

We agree that our original phrasing overstated the level of host control supported by the data. We have revised the manuscript throughout to avoid implying active or complete host regulation and instead frame these patterns as consistent with host involvement. While symbiont gene expression showed little change, the activation of specific host genes suggests that fluctuations in symbiont titre may be influenced by host physiology, however, direct evidence of active host control has yet to be demonstrated. We have therefore tempered the wording accordingly, including revising the highlighted sentence to "This rapid rebound *could indicate a host-assisted* symbiont recovery during a key developmental transition" (line 128-129), with additional changes on lines 35, 89-90, 123, 136-137 and 316.

Why were metabolomics performed only on late pupae, and symbiont titre measured only in third-instar larvae? It would strengthen the manuscript if the authors could justify these choices, rather than a collection of techniques applied to different samples without a connected story.

These are important points to clarify, thank you for bringing them to our attention. The sampling decisions were intentional and biologically motivated. Metabolomics was performed on late-stage pupae as this is when the cuticle begins to melanise and sclerotise so we would expect the presence and metabolism of tyrosine at this stage. Third instar larvae were selected for symbiont titre measurements because they showed the lowest variation (i.e., the narrowest standard error bars across our dataset) compared to other developmental stages. This reduced the likelihood that natural variation would obscure overall trends and increased our ability to detect treatment effects. These details have been added to the methods sections on lines 504-506 and 493-496.

Line 385: "Aposymbiotic colonies were … allowed to re-queen before experimentation" – were they de-queened during the heat-treatment to remove Westeberhardia? And were the aposymbiotic colonies introduced with only one queen instead of multiple queens as the natural aposymbiotic colony?

Colonies were not de-queened before or during heat treatment. Following treatment, all existing alates (unmated, winged queens) were removed, leaving only established mated queens (identified by the absence of wings). When new alates emerged post-treatment, the original mated queens were removed, and the alates were allowed to mate. This is how we ensured queens involved in the experiment were not subjected to the heat treatment. We have clarified this in the Methods (lines 394-395).

Lines 442-444: What were the insects being fed here? Earlier it was mentioned the standard colonies were fed cockroaches (once weekly), and fruit flies (twice weekly). Were a combination of cockroaches and fruit flies being used in control and low protein diet? Please also specify the sources of the insects provided, and how they were fed – were whole fresh insects offered, or were they cut open, etc.?

These are important points which we have now clarified in the methods. The cockroaches were provided in the form of one head or two legs, and we delivered five fruit flies whole to each colony. In the weeks that the low protein colonies received insects, these were provided in exactly the same way as the control colonies (cockroaches once and flies twice), so that only the frequency of receiving protein meals was varied. This is now explicitly stated in lines 453-455.

Lines 444-445: "each symbiotic colony was split in two" - How many origin symbiotic colonies were used in this experiment? Were all the 6 groups (3 diet, 2 symbiotic status) derived from a single original colony, or were multiple colonies included?
Sample size is missing in some experiments, e.g., metabolomics.

There were originally 7 different source colonies that were collected from Regensburg and these were used as the 7 replicates in our study, i.e., source colony 1 was split into symbiotic and aposymbiotic and then each split again for each diet. This maintained sufficient genome variation between replicates, while maintaining genetic similarity across treatments. Added in brief to line 455-456. Sample sizes were indeed missing for Figure S6 on metabolomics and throughout the

gene expression figures (Fig. 3, Fig. 4A-D, Fig. 5A, Fig. 6A, Fig. S3, Fig. S5), these have all now been added and can be found in the figure captions.

Minor comments:

The abstract does not mention methods. It would be helpful to include the techniques used in the study to clarify the scope.
We thank the reviewer for drawing our attention to this and have now added some methodological details in lines 28-33.

Line 31: spell out "Cand." as it was first time mentioned.
Fixed

Line 79: change to carbohydrate-to-protein
Fixed

Lines 103-109: some "p" were italicized, some were not
All italicised now

Lines 126-132: add in statistical support
Added into text and into supplementary material, Table-S1-ModelResults, sheet 6-Symbiont-Density. A correction of one p value has also been made to Figure 2A resulting from typo, but the overall significance and trend remain the same.

Figure 3: Since each bar represents upregulated transcripts within the comparison, it would be helpful to stack the dark blue and light blue pairs together for each caste or life stage (i.e., upregulated and downregulated side-by-side).
We thank the reviewer for this suggestion and apologise for the misleading caption. To clarify, the bars do not represent up- versus downregulated transcripts in a directional sense, but rather transcripts that are more highly expressed in one condition relative to the other. Presenting them as stacked up/down pairs could therefore imply a directional regulatory interpretation that is not intended. We have retained the current visualisation as we feel it reflects this distinction more accurately and have clarified this in the figure legend to avoid confusion.

Line 338 and 358: Change the dash to emdash
Fixed

Lines 406: What is the read length?
100bp, now added to methods.
