# Supplementary material for: Metabolic plasticity supports a flexible nutritional symbiosis in Cardiocondyla ants
Source: Nat Commun. 2026 Aug 21;17:10002. doi: 10.1038/s41467-026-76801-z (PMC13590504; doi:10.1038/s41467-026-76801-z)
Supplement: Supplementary file 3 [file 41467_2026_76801_MOESM2_ESM.pdf]

Descriptions of Additional Supplementary Files

Supplementary Data 1- ModelResults. Results from the statistical modelling performed for the phenotypic analyses.

Supplementary Data 2- Sample-Metadata. Metadata pertaining to each specific sample.

Supplementary Data 3- DESeq2-DEGs-ComparisonAnnotated. Genes identified through DESeq2 differential gene expression analysis annotated using interpro, KEGG, PANTHER and GO.

Supplementary Data 4- STAR-Counts. Raw gene expression counts generated by STAR and used in DESeq2 analysis (sheet 1 for C. obscurior host and sheet 2 for Westeberhardia)

Supplementary Data 5- Counts-vst-normalised. Data frame use to generate gene expression figures, counts are normalised using variance stabilising transformation within DESeq2 package.

Supplementary Data 6- ColonyFitness. Phenotypic data from diet treatment experiments including colony mortality, pupae size and brood production.

Supplementary Data 7- qPCR-SymbiontDensity. Raw and logged qPCR results.

Supplementary Data 8- MetabolomicsLifestagesCalc. Raw and processed metabolomic data for across life stages.

Supplementary Data 9- MetabolomicsDietTreatmentsCalc. Raw and processed metabolomic data collected during the diet treatments.

Supplementary Data 10- CuticleMeasurements. Raw and processed measurements of cuticle thickness, investment and colouration.

Supplementary Data 11-C-obscurior-Annotations. Host Cardiocondyla obscurior genome annotation file.

Supplementary Data 12-C-obscurior-COG. Host Cardiocondyla obscurior genome COG annotations.

Supplementary Data 13- Westeberhardia Annotations. Table containing standard gene annotations from PROKKA for the Westeberhardia symbiont.

Supplementary Data 14- oPools. Oligonucleotide pools generated for RNA in situ hybridisations presented in main and supplementary figures.
