# Supplementary material for: Metabolic plasticity supports a flexible nutritional symbiosis in Cardiocondyla ants
Source: Nat Commun. 2026 Aug 21;17:10002. doi: 10.1038/s41467-026-76801-z (PMC13590504; doi:10.1038/s41467-026-76801-z)
Supplement: Supplementary file 4 [file 41467_2026_76801_MOESM1_ESM.docx]

**Supplementary Information**

**Includes:**

Supplementary methods for metabolomics work. Provided below.

Supplementary Figures S1-S6. Provided below.

Supplementary data 1-14 provided as separate excel files.

**Supplementary methods**

*Sample Processing*

To measure amino acid levels, late-stage pupae were frozen, crushed under liquid nitrogen, and stored in 500 µL of 80% HPLC-grade methanol before being sent to the Max Planck Institute in Jena, Germany, for processing. Two negative control extracts without ants were included. Due to sample limitations, amino acids were quantified only in the control and low-protein diet treatments. A 50-µL aliquot of the sample extract was diluted with 50 µL of an aqueous mix of ^13^C,^15^N-labelled amino acid standards (Aldrich, cat. no. 487910).

*Internal standards*

Final concentrations of the isotope-labeled amino acid standards:

887 fmol/μL ^13^C_3_,^15^N-Ala;

145 fmol/μL ^13^C_6_,^15^N_4_-Arg;

456 fmol/μL ^13^C_4_,^15^N-Asp;

399 fmol/μL ^13^C_5_,^15^N-Glu;

2050 fmol/μL ^13^C_2_,^15^N-Gly;

31 fmol/μL ^13^C_6_,^15^N_3_-His;

166 fmol/μL ^13^C_6_,^15^N-Ile;

331 fmol/μL ^13^C_6_,^15^N-Leu;

56 fmol/μL ^13^C_6_,^15^N_2_-Lys;

350 fmol/μL ^13^C_5_,^15^N-Met;

114 fmol/μL ^13^C_9_,^15^N-Phe;

254 fmol/μL ^13^C_5_,^15^N-Pro;

306 fmol/μL ^13^C_3_,^15^N-Ser;

286 fmol/μL ^13^C_4_,^15^N-Thr;

82 fmol/μL ^13^C_9_,^15^N-Tyr;

325 fmol/μL ^13^C_5_,^15^N-Val

Chromatography

Samples were kept at 10°C, and 2 µL were injected into an UltiMate™ 3000RS UHPLC system (Thermo Scientific) with a Zorbax Exclipse C18 column (Agilent), using acidified water and methanol in gradient mode^1^.

*Instrument setup*

- Solvent-Rack/Degasser:	Thermo SRD-3600 Solvent Rack
- LC-Pump/GC-Oven:	Thermo HPG-3400RS Pump
- Autosampler:	Thermo WPS-3000TRS Autosampler
- Column Compartment:	Thermo TCC-3000RS Column Compartment
- Column:	Zorbax Eclipse XDB-C18 (3.0 × 50 mm, 1.8 µm; Agilent)
- *Gradient conditions*
- column temperature:	42°C
- solvent A:	0.05% formic acid, 0.1% acetonitrile in water
- solvent B:	methanol
- gradient:	0.0 min	1% B (flow 0.25 mL min^-1^)

1.0 min	1% B (flow 0.5 mL min^-1^)

2.7 min	100% B (flow 0.5 mL min^-1^)

3.9 min	1% B (flow 0.25 mL min^-1^)

4.9 min	1% B (flow 0.25 mL min^-1^)

Mass spectrometry

Natural amino acid isomers and internal standards were detected via EVOQ™ Elite triple-quadrupole MS (Bruker) with heated electrospray ionisation in positive mode (Spray voltage: 4500 V; Cone gas: 35 AU; Cone temp: 350°C; Probe gas: 50 AU; Probe temp: 500°C; Nebulizer gas: 60 AU). Samples were analysed in multiple reaction monitoring (MRM) mode as described in Schäfer et al. (2016)^1^ (Supplementary Table 1).

*Data analysis*

Post-run analysis was done using Bruker MS Workstation (v8.2.1) and concentrations were calculated using internal standards (details provided in main text).

**Supplementary figures**

**Figure S1. Brood production in response to diet treatments in aposymbiotic vs symbiotic colonies.** (A) Larvae and (B) pupae production under control (dark/light blue), low-protein (dark/light pink), and no-protein (dark/light orange) diets in symbiotic vs. aposymbiotic colonies. Statistical significance was assessed using generalised linear mixed-effects models fitted with a negative binomial distribution and colony identity as a random effect, followed by tukey-adjusted two-sided Wald z-tests for pairwise comparisons (n = 7 biological replicates per group, *p* values based on glmer modelling show significant differences between slopes of colony type).

**Figure S2. Cuticle investment in response to colony types.** Cuticle proportion measured as a ratio of total volume of cuticle to total ant volume. (A) Cuticle investment across diets and colony types after 12 weeks of treatment: blue (symbiotic on control diet), light blue (aposymbiotic on control diet), magenta (symbiotic on low protein diet), light pink (aposymbiotic on low protein diet), dark orange (symbiotic on no protein diet), light orange (aposymbiotic on no protein diet); (B) Cuticle investment grouped by colony type irrespective of diet treatment. Note samples size are small due to brood production constraints (see main manuscript) and vary from N=4 to N=1. Linear regression analysis was performed on all data shown, and statistical significance was assessed using two-sided t-tests derived from the linear model coefficients. No multiple-comparison correction was applied. No significance was found.

**Figure S3. Heatmap of Westeberhardia gene expression across host life stages.** Colour intensity refers to the level of gene expression normalised using variance stabilising transformation, numbers are arbitrary but higher values indicate greater gene activity. No significant differences in *Westeberhardia* gene expression across host life stages or castes were reported by DESeq2 (n=3 biological replicates per life stage or caste).

**Figure S4. Myogenesis-regulating glycosidase which can produce β-D-galactose/β-D-glucose.** Strong gene expression in the gut tissue of (A) early larval instar 2 and (B) larval instar 3. (C) Expression of an organic cation transporter gene found localised to bacteriocytes of symbiotic larvae. RNA-FISH using HCR, *Westeberhardia* 16S ribosomal mRNA shown in yellow, and host genes are in magenta.

**Figure S5. Functionally enriched genes across life stages.** Bar graph showing functionally enriched groups using INTERPRO annotations, further functional annotation was estimated using other annotation tools such as GO, KEGG and PANTHER. Functionally enriched genes were not identified in the queen caste (n=3 biological replicates per life stage or caste).

**Figure S6. Tyrosine and other amino acid level across life stages and diet treatments.** (A) Amino acid level measured as nmol per ant across life stages (n=9 biological replicates). L2: larval instar 2, EL3: early larval instar 3, LL3: late larval instar3, PW: white pupae, PB: brown pupae, NW: new worker, OW: old worker. Transition between PW and PB are highlighted in red or green to show an increase (green) or a decrease (red) in level of amino acid. Tyrosine is highlighted in green as the only amino acid to increase during pupation. Data are presented as mean values +/- SEM. (B) Amino acid level measured as nmol per ant averaged across the 12 weeks of the diet treatment for the control and low-protein diet (n = 10 biological replicates, raw data is presented on top of box plots). There was not enough brood in the no-protein diet for sampling. Plots highlighted in grey show amino acids significantly higher in symbiotic colonies across the control diet, highlight in orange are the amino acids higher in symbiotic colonies across the control diet and the low-protein diet (tyrosine only). Linear regression analysis was performed on all data shown, and statistical significance was assessed using two-sided t-tests derived from the linear model coefficients. No multiple-comparison correction was applied. Non-highlighted plots showed no significant values. Model results can be found in Supplementary Data 1.

**Supplementary References**

1.	Schäfer, M., Brütting, C., Baldwin, I. T. & Kallenbach, M. High-throughput quantification of more than 100 primary- and secondary-metabolites, and phytohormones by a single solid-phase extraction based sample preparation with analysis by UHPLC–HESI–MS/MS. *Plant Methods* **12**, 30 (2016).
